# Comparative DNA Methylome of Phytoplasma Associated Retrograde Metamorphosis in Sesame (*Sesamum indicum* L.)

**DOI:** 10.3390/biology11070954

**Published:** 2022-06-23

**Authors:** Pratima Verma, Amrita Singh, Supriya Purru, Kangila Venkataramana Bhat, Suman Lakhanpaul

**Affiliations:** 1 Department of Botany, University of Delhi, New Delhi 110007, India; vpratima96@gmail.com; 2 Department of Botany, Gargi College, University of Delhi, New Delhi 110049, India; amrita.botany@gmail.com; 3 ICAR-NAARM, Rajender Nagar, Hyderabad 500030, India; supriya@naarm.org.in; 4 ICAR-National Bureau of Plant Genetic Resources, New Delhi 110012, India; kvbhat2001@yahoo.com

**Keywords:** *Sesamum indicum* L., DNA methylation, Whole Genome Bisulfite Sequencing (WGBS), phytoplasma, phyllody, little leaf and McrBC

## Abstract

**Simple Summary:**

Sesame (*Sesamum indicum* L.) is an important oilseed crop that is well known for its highly nutritious oil content. Due to its high oil content and nutritional properties, sesame is also known as the ‘queen of oilseeds’. Phytoplasma-associated diseases such as phyllody and little leaf are critical threats to sesame cultivation worldwide. Sesame phyllody is the leading biotic constraint drastically affecting sesame productivity and resulting in yield losses of up to 80% in major sesame-producing countries in the world. In this study, we explored the role of DNA methylation in phytoplasma infection. Whole Genome Bisulfite Sequencing and a Quantitative analysis of DNA methylation using real-time PCR (qAMP) revealed an alteration of methylation pattern upon phytoplasma infection. Few selected development and defense-related loci were either hypo- or hypermethylated. We hereby report the first methylome study in healthy and phytoplasma-infected sesame. Our study provides fundamental insights into the role of DNA methylation in the symptom development of phytoplasma infection in sesame plants.

**Abstract:**

Phytoplasma-associated diseases such as phyllody and little leaf are critical threats to sesame cultivation worldwide. The mechanism of the dramatic conversion of flowers to leafy structures leading to yield losses and the drastic reduction in leaf size due to Phytoplasma infection remains yet to be identified. Cytosine methylation profiles of healthy and infected sesame plants studied using Whole Genome Bisulfite Sequencing (WGBS) and Quantitative analysis of DNA methylation with the real-time PCR (qAMP) technique revealed altered DNA methylation patterns upon infection. Phyllody was associated with global cytosine hypomethylation, though predominantly in the CHH (where H = A, T or C) context. Interestingly, comparable cytosine methylation levels were observed between healthy and little leaf-affected plant samples in CG, CHG and CHH contexts. Among the different genomic fractions, the highest number of differentially methylated Cytosines was found in the intergenic regions, followed by promoter, exonic and intronic regions in decreasing order. Further, most of the differentially methylated genes were hypomethylated and were mainly associated with development and defense-related processes. Loci for STOREKEEPER protein-like, a DNA-binding protein and PP2-B15, an F-Box protein, responsible for plugging sieve plates to maintain turgor pressure within the sieve tubes were found to be hypomethylated by WGBS, which was confirmed by methylation-dependent restriction digestion and qPCR. Likewise, serine/threonine-protein phosphatase-7 homolog, a positive regulator of cryptochrome signaling involved in hypocotyl and cotyledon growth and probable O-methyltransferase 3 locus were determined to be hypermethylated. Phytoplasma infection-associated global differential methylation as well as the defense and development-related loci reported here for the first time significantly elucidate the mechanism of phytoplasma-associated disease development.

## 1. Introduction

Sesame (*Sesamum indicum* L.) is an important oilseed crop well known for its highly nutritious edible oil and is cultivated in tropical and subtropical areas of Asia and Africa. The seed oil content ranges from 50–60% [1]. Sesame oil is rich in natural antioxidants such as sesamolin, sesamin and sesamol. In addition, sesame seeds are a rich source of protein (18–25%), carbohydrates (13.5%), vitamins and minerals [2]. Due to its high oil content and nutritional properties, sesame is also known as the ‘queen of oilseeds’ [3].

India is the third leading producer of sesame seeds after Sudan and Myanmar and has the world’s largest area under sesame cultivation. A low harvest index, seed shattering, lack of varieties resistant to biotic and abiotic stresses and indeterminate growth habits are some of the reasons leading to low sesame productivity per hectare [4,5]. Sesame phyllody is the leading biotic constraint drastically affecting sesame productivity, resulting in yield losses of up to 80% in major sesame-producing countries in the world [6].

Sesame plants infected with phytoplasma show many conspicuous symptoms such as phyllody, witches broom, fasciation, little leaf, etc. The most prevalent symptom, sesame phyllody, is marked by retrograde metamorphosis, i.e., a reversion of the reproductive phase to the vegetative phase wherein flowers are replaced by proliferating leafy structures. In severe cases, the proliferation of leaves and branches becomes so substantial that it gives the appearance of a ‘witches’ broom’ to the affected plants [7]. Little leaf symptom is marked by a smaller than average size of leaves and, the plants usually do not enter the reproductive phase. Fasciation, yet another symptom associated with phytoplasma, is commonly manifested as the enlargement and flattening of the stem in sesame plants.

The mechanism behind phytoplasma-induced phyllody has been attributed to the interaction of effector molecules, i.e., SAP54 and SAP11 with the host plant transcription factors (TFs) viz., MADS Box TFs and TCP (TEOSINTE BRANCHED1 (TB1), CYCLOIDEA (CYC), PROLIFERATING CELL NUCLEAR ANTIGEN FACTOR1 (PCF1) and (PCF2) transcription factors [8,9,10,11,12,13,14]. The recently reported phytoplasma effector, SAP05, has been described to bind to SPL and GATA transcription factors, mediate their degradation, slow plant aging, and multiply vegetative tissues and shoots [15].

In essence, phytoplasma-associated symptoms are results of dramatic alterations in the normal developmental program of the host plants that often result in economic yield losses.

However, the mechanism involved and the level at which the genetic regulation of the host plants is adversely affected remains far from understood and is increasingly gaining attention.

Researchers have begun to explore how plants respond to phytoplasma and the precise levels of gene regulation such as epigenetic, transcriptional, post-transcriptional and translational levels. DNA methylation is one of the epigenetic mechanisms controlling gene expression which involves the chemical modification of the 5′-a position of cytosine bases of DNA by the addition of a methyl group (-CH_3_) [16]. Developmental abnormalities have been reported in plants as a consequence of DNA methylation disruption [17]. The role of DNA methylation in biotic stress has been frequently reported in plant–pathogen interactions. For example, considerable differences were observed in cytosine methylation in the seedlings and adult plants of the rice cultivar Wase Aikoku 3 and higher methylation levels render the adult plants resistance against the blight pathogen *Xanthomonas oryzae* pv. *oryzae* [18]. The bacterial pathogen *Pseudomonas syringae* pv. *tomato* DC3000 induced DNA hypomethylation in leaf tissues of *Arabidopsis thaliana* via an active demethylation process [19]. The first genome-wide single-nucleotide-resolution DNA methylome was studied by Dowen et al. [20] which revealed that DNA demethylation increased *Arabidopsis thaliana* resistance to the bacterial pathogen *Pseudomonas syringae* pv. *tomato*. The enrichment of differentially methylated cytosines was found in gene-rich regions of the genome. DNA demethylation in transposable elements during antibacterial defense led to their transcriptional up-regulation [20,21].

The epigenetic response in plants upon phytoplasma infection was explored in mulberry (*Morus multicaulis* Perr.) [22], while the methylation level of infected leaves was not significantly different from that of healthy leaves. It was found that methylation of the G-type lectin serine/threonine protein kinase (MuGsSRK) gene was decreased, but its expression level was increased in leaves infected with the pathogen or treated with G. Salicylic Acid (SA). Genome-wide bisulfite sequencing (WGBS) analysis in *Vitis vinifera* L. revealed the presence of DNA methylation markers that differ between spontaneous and plant-dependent recovery and healthy leaves [23].

In *Paulownia fortunei*, Amplified Fragment Length Polymorphism (AFLP) and Methylation-Sensitive Amplification Polymorphism (MSAP) results showed the occurrence of paulownia Witches’ Broom (PaWB) related to changes in DNA methylation level [24]. Later, WGBS and RNAseq techniques were used to study DNA methylation and gene expression changes between healthy and phytoplasma-infected *Paulownia Fortunei* seedlings. Compared with healthy seedlings, DNA methylation levels increased after phytoplasma infection, and mCHH methylation was majorly altered [25]. Recently, N6-methyladenosine (m^6^A-seq) sequencing was used to determine methylation levels in phytoplasma-infected paulownia in combination with a transcriptome analysis to screen for PaWB-associated differentially expressed genes. M^6^A modification levels were higher in seedlings infected with phytoplasma that causes Paulownia witches’ broom (PaWB) when compared to infected seedlings treated with methyl methanesulfonate (MMS) [26].

Hypermethylation of the regulatory region of *SIDEFICIENS* (*SIDEF*) gene was reported in tomato plants infected with stolbur phytoplasma isolate PO [27]. The downregulation of demethylase genes and genes coding for chromomethyltransferases (CMT) was also observed in stolbur PO-infected tomato plants. 

Thus, the role of DNA methylation in phytoplasma symptom development has been suggested in a few crop species; however, comprehensive studies on whole genome cytosine methylation are scarce and completely lacking in sesame. In the present study, we report the DNA methylation profiling of healthy and phytoplasma-infected plants showing symptoms of phyllody and little leaf using a whole-genome bisulfite sequencing (WGBS) approach to investigate the possible role of phytoplasma-induced epigenetic regulation as the mechanism of symptom development. The information provided will assist in resolving the epigenetic processes underlying the responses of sesame to phytoplasma infection, and thus provide important clues for further studying the disease resistance mechanism for molecular breeding.

## 2. Materials and Methods

### 2.1. Plant Material

**Figure 1:**
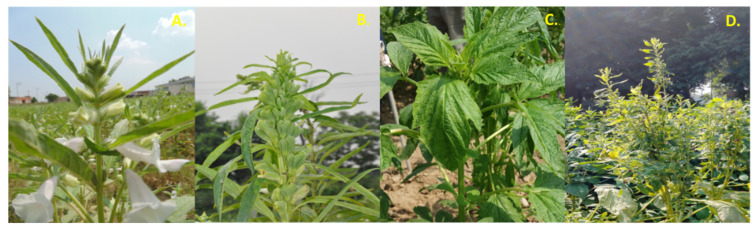
*Sesamum indicum* L. plants (**A**) Asymptomatic flowering twig (HF) (**B**) Symptomatic twig showing phyllody (I1) (**C**) Asymptomatic vegetative twig (H1) (**D**) twig showing little leaf symptom (LL).

Asymptomatic vegetative (H1), asymptomatic flowering (HF), Phyllody (I1) and Little Leaf (LL)-affected *Sesamum indicum* (L.) cv Sekhar plant samples were collected from fields in Baghpat, Uttar Pradesh, India (Figure 1). The samples were frozen in liquid nitrogen (LN_2_) and stored at −20 °C until use. All the samples were collected in triplicate to be used as biological replicates. 

### 2.2. DNA Extraction and Quantification

Total genomic DNA was extracted from asymptomatic and symptomatic sesame samples and also from the phytoplasma-affected *Catharanthus roseus* sample by employing the CTAB (Cetyl trimethylammonim bromide) method [28]. The assessment of the quality and quantity of extracted DNA was performed using agarose gel (1.8%) electrophoresis and Nanodrop (Thermo Scientific™ Multiskan™ GO, Waltham, MA, USA).

### 2.3. Phytoplasma Detection Using Nested-PCR

The presence of phytoplasma in the symptomatic sesame sample was confirmed using the phytoplasma-specific universal 16S rDNA primer pair P1/P7 [29], followed by nested PCR using primer pair R16F2/R2n [30]. *Catharanthus roseus* DNA samples previously confirmed for the presence of phytoplasma were used as the positive control. The amplified 16SrRNA gene product was electrophoresed on 1.2% agarose gel, gel extracted and sequenced with 2X coverage. The sequence obtained was analysed for homology using NCBI BLASTn.

### 2.4. DNA Library Construction and Whole Genome Bisulfite Sequencing

The genomic DNA was sheared using the Covaris sonicator and 100–300 bp fragments were obtained. DNA purification was conducted using AMPure beads. Illumina TruSeq kit was used to end repair the purified fragmented DNA and for ‘A’ tailing. TruSeq DNA adapters containing 5-methylcytosines were ligated to the sheared DNA and the ligated DNA was further purified using AMPure beads. Bisulfite treatment of the ligated DNA fragments was carried out using EZ DNA Methylation-Gold Kit (Zymo Research) following the manual’s instructions. The bisulfite treated DNA fragments were PCR amplified using the PCR primer cocktail (Illumina TruSEq kit). The bisulfite libraries, thus constructed, were purified using AMPure beads and subjected to quality testing using Bioanalyzer (Agilent 2100). The library (~10 pM) was sequenced using the Illumina HiSeq 2500 machine for 90 cycles in the paired-end mode.

### 2.5. Quality Check of Reads

FastQC (version 0.11.5, https://www.bioinformatics.babraham.ac.uk/projects/fastqc/) (accessed on 30 May 2017) was used to examine the quality of the sequenced reads. The parameters included were base quality score distribution, sequence quality score distribution, average base content per read and GC distribution in the reads. The adapters and reads of low quality were removed using TrimGalore (https://www.bioinformatics.babraham.ac.uk/projects/trim_galore/) (accessed on 5 June 2017) which uses Cutadapt (https://github.com/marcelm/cutadapt) (accessed on 5 June 2017) and FastQC (http://www.bioinformatics.babraham.ac.uk/projects/fastqc/) (accessed on 5 June 2017) tools.

### 2.6. Mapping of High-Quality Reads

The reads were aligned to the reference genome *Sesamum indicum S_indicum_v1.0,* RefSeq: GCF_000512975.1 (Submitter: Oil Crops Research Institute of the Chinese Academy of Agricultural Sciences) using BSMAP v 2.90 (whole genome bisulfite sequence MAPping program) [31]. Methratio (https://github.com/genome-vendor/bsmap/blob/master/methratio.py) (accessed on 7 June 2017), a python script, was used to extract methylation ratios from BSMAP mapping results. *p*-values were calculated to determine true methylated cytosines and only the cytosines with a *p*-value < 0.0025 were considered to be truly methylated.

### 2.7. Calculation of Absolute and Relative Methylation Percentages

Absolute and relative methylation percentages were calculated using the following formulae:

$$\mathrm{Absolute}\;\mathrm{Methylation}\;\mathrm{Percentage}\left(\%\right)=\frac{\mathrm{Total}\;\mathrm{methylation}\;\mathrm{levels}\;\mathrm{of}\;\mathrm{mCs}\;\times \;100}{\mathrm{Total}\;\mathrm{number}\;\mathrm{of}\;\mathrm{methylated}+\mathrm{unmethylated}\;\mathrm{Cs}}$$

$$\mathrm{Relative}\;\mathrm{Methylation}\;\mathrm{Percentage}\left(\%\right)=\frac{\mathrm{Total}\;\mathrm{methylation}\;\mathrm{levels}\;\mathrm{of}\;\mathrm{mCs}\;\times \;100}{\mathrm{Total}\;\mathrm{sequence}\;\mathrm{length}\;\mathrm{of}\;\mathrm{calculated}\;\mathrm{region}}$$

### 2.8. Descriptive Statistics on Samples

MethylKit, an R package developed by [32] for DNA methylation analysis, was used for whole methylome characterization. Percent methylation distribution plots were obtained for healthy and infected samples in all the three sequence contexts (CG, CHG and CHH) using the script getMethylationStats. Plots showing read coverage per base were obtained using the command getCoverageStats. 

### 2.9. Identification and Annotation of Differentially Methylated Bases

Differentially methylated cytosines (DMCs) at each sequence context (CG, CHG and CHH) were extracted using the get.methylDiff (Object difference = 25, q value = 0.01, type = hypo/hyper) function based on the following default parameters of methylKit: bases with q-value < 0.01, bases with % methylation difference > 25% and type of differential methylation (hypo-/hypermethylation). Differentially methylated cytosines (DMCs) were placed into their genomic contexts to analyze their biological significance using the genomation package (https://bioconductor.org/packages/release/bioc/html/genomation.html) (accessed on 21 June 2017) of methylKit. The reference genome used was *Sesamum indicum S_indicum_v1.0*, RefSeq: GCF_000512975.1 (Submitter: Oil Crops Research Institute of the Chinese Academy of Agricultural Sciences).

### 2.10. CpG Island Prediction, Gene Structure Prediction and Primer Designing 

Four loci, namely F-box protein PP2-B15 (FBOX), STOREKEEPER protein-like (STKL), Probable O methyltransferase 3 (OMT) and serine/threonine-protein phosphatase 7 long form homolog (STP) were selected due to their significant role in development and defense related processes for detailed analysis. FBOX and OMT were found to be differentially methylated only in little leaf-affected samples and STP, and STKL were found to be differentially methylated in phyllody as well as little leaf samples. CpG island prediction was carried out using MethPrimer http://www.urogene.org/cgi-bin/methprimer/methprimer.cgi (accessed on 25 May 2021) [33]. CpG island-specific primers were designed using Primer Blast Tool NCBI [34]. The amplicon was analyzed for McrBC restriction sites (5′. . .Pu^m^C (N_40–3000_) Pu^m^C. . .3′). The gene structure predictions were made using GSDS 2.0 (http://gsds.gao-lab.org/) (accessed on 16 January 2022) [35].

### 2.11. Methylation Dependent Restriction Digestion and Chop-PCR

A total of 2.5 µg of the isolated genomic DNA of healthy and phytoplasma-infected samples were homogenized by repeatedly passing the samples through a 25.5 gauge needle attached to a 1 mL syringe to reduce the average size of the DNA fragment to ~4–5 kb. Then, 500 ng of this sheared DNA was digested with McrBC enzyme (NEB) following the manufacturer’s instructions. In mock digestions, the McrBC enzyme was replaced with Milli-Q water. The mix was incubated for 16 h at 37 °C. Heat inactivation of the restriction enzyme was performed by incubating the reaction at 65 °C for 20 min in water bath. Ten ng of four different templates, i.e., HM-healthy mock, HD-healthy digested, IM-infected mock, ID-infected digested, were PCR amplified. Healthy genomic DNA containing no component of McrBC was used as the positive control.

### 2.12. Quantitative Analysis of DNA Methylation Using Real-Time PCR (qAMP)

In order to test the hypo or hypermethylation status of selected loci, Quantitative PCR was performed using the GoTaq^®^ qPCR Master Mix (Promega) according to the manufacturer’s suggested conditions for use with the LightCycler^®^ 480 Instrument (Roche Applied Science). Reactions were mixed in a total volume of 20 µL with 0.1 ng of template DNA and 0.2 µM of forward and reverse primers [34]. The relative amplification was calculated using the ΔΔCt method. To calculate ΔCt, the Ct of the unmethylated control (Actin) was subtracted from the Ct of the target region. To obtain ΔΔCt, the ΔCt value of the untreated sample was subtracted from the ΔCt value of the enzyme-treated sample, i.e., ΔΔCT = [CT (digested) − CT (mock)] [36], and the percentage of methylation of a given site was described using the formula % me = 100(1 − e^−0.7(ΔΔCt)^) [37]. Each sample was screened in triple technical replicates. Non-specific amplification was monitored using the melting curve analysis of each reaction.

## 3. Results

### 3.1. Detection and Classification of Phytoplasma from Symptomatic Sesame

Amplicons of approximately 1.2 kb were obtained after nested-PCR confirming the presence of phytoplasma in the sesame plants showing Phyllody (I1) and Little Leaf (LL) symptoms. No amplicon was observed in the case of asymptomatic vegetative (H1) and asymptomatic flowering (HF) samples. The amplicon sequence showed 99% identity with the 16SrRNA gene sequence of Onion yellows phytoplasma strain M (OY-M), GenBank Accession Number AP006628 which belongs to the 16SrI-B (sub) group.

### 3.2. Profiling of Genome Wide DNA Methylation

Healthy flowering (HF), phyllody affected (I1), healthy vegetative (H1) and little leaf-affected (LL) samples were subjected to Whole Genome Bisulphite Sequencing (WGBS), with two replicates each. H1 was used as the control for LL and HF was used for I1. Scatter plots of % methylation values between the samples confirmed their high repeatability (Figure S1 and Table S1).

To obtain genomic DNA methylation profiles of phyllody and little leaf-affected sesame plants, BS-seq was performed. More than 153 million paired-end reads were generated from four samples. After removing low-quality and adapter-polluted reads, the rate of clean reads was above 97%. Unique reads were then mapped to the sesame reference genome sequence. The mapped percentages were 82.17%, 84.91%, 84.44% and 85.10% for HF, I1, H1 and LL, respectively. Amongst all the samples, the highest mapping efficiency was obtained in little leaf (LL) (Table 1).

The read coverage histogram plots of all samples showed no secondary peaks in any of the three contexts, thus indicating the accurate determination of % methylation scores without any PCR duplication bias (Figure S2).

The absolute methylation percentage of all the samples ranged between 40–46%. Almost twice the relative methylation was observed in Phyllody (I1) when compared to healthy flowering (HF) although comparable relative methylation was observed in Little Leaf (LL) and healthy vegetative (H1) (Table S2).

**Figure 2:**
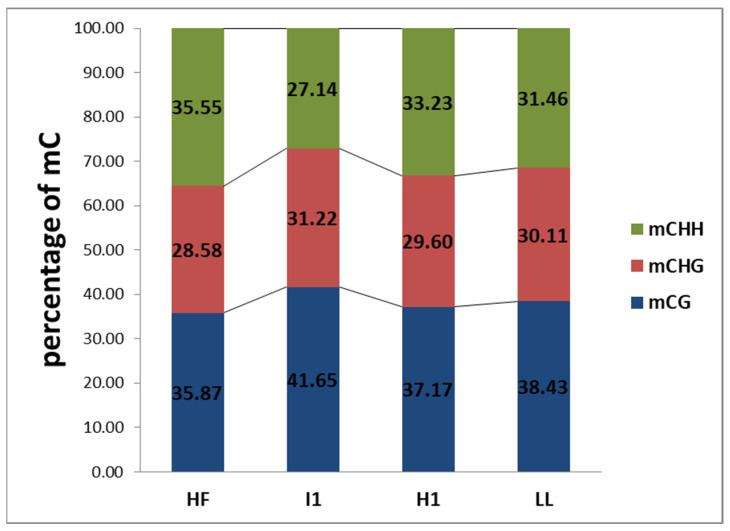
The percentage of Methylcytosines (mCs) in different sequence contexts identified in healthy flowering (HF), phyllody (I1), healthy vegetative (H1) and little leaf (LL) sesame samples.

Among the three types (mCG, mCHG, mCHH), the percentage of mCs ranged between 27–40%. CHH methylation levels were higher in HF and H1 when compared to I1 and LL, respectively. CG and CHG methylation levels were lower in HF and H1 than in I1 and LL (Figure 2).

### 3.3. Distribution of DNA Methylation 

**Figure 3:**
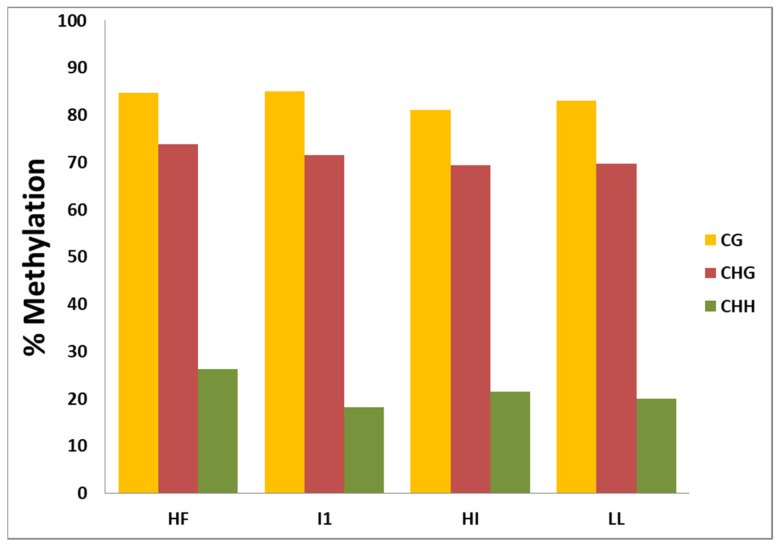
Percentage of Methylcytosines (mCs) over total CT count in CG, CHG and CHH contexts. HF-healthy flowering, I1-Phyllody-affected H1-healthy vegetative and LL-little leaf sesame samples.

After the reads were mapped to the sesame reference genome sequence, the cytosine (include CG, CHG, CHH) methylation rates calculated using mCs over total CT counts was found to be nearly comparable in the CG and CHG context among the samples. The CHH methylation level in HF (26.26%) was significantly higher than that in I1 (18.22%) (Figure 3).

To compare the methylation regions of different samples, DMRs were screened out, and the methylation regions that potentially regulate gene expression were further analysed. Three regions (1000-bp sequence upstream of the coding gene (up1k), the gene body sequence and 1000-bp sequence downstream of the gene body (down1k)) showed differences between the three samples. In all the samples, the fraction of mCs was higher in the gene body in the CG context when compared to the CHG and CHH context approx. 0.35 fractions of mCs in HF, H1 and LL but decreased to 0.30 in I1. The CG methylation level in the gene body was significantly higher than in the 1 kb upstream (up1k) and downstream (down1k) regions of transcription start and stop sites, respectively. The percentage of mCs increased as we moved 1 kb upstream or downstream from start and stop sites correspondingly.

A similar trend was observed in the CHG context, though the fraction of mCs in the gene body was lower in healthy vegetative (H1) and little leaf (LL) than 1 kb upstream (up1k) and downstream (down1k), whereas in healthy flowering (HF) it was almost equal to the 1 kb upstream and downstream regions. Contrarily, the mCs in the gene body was higher in Phyllody (I1) than 1 kb upstream (UPS) and downstream (DS) regions.

**Figure 4:**
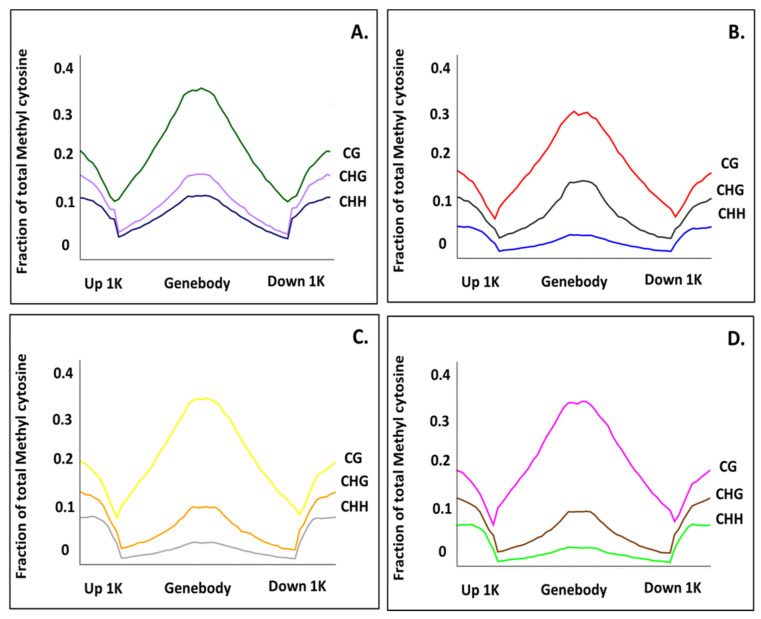
Line plots showing methylation levels in gene body, upstream and downstream flanking regions (up to 1 Kb) in (**A**) healthy flowering (HF) (**B**) phyllody affected (I1) (**C**) healthy vegetative (H1) (**D**) little leaf affected (LL) samples in CG (Top most line), CHG (Middle line) and CHH contexts (lower line).

In the CHH context, the fraction of mCs in the gene body was comparable to the 1 kb upstream and downstream regions, i.e., ~0.12 fractions in the case of healthy flowering (HF). However, in the healthy vegetative (H1), little leaf (LL) and phyllody-affected sample (I1), the fraction of mCs in the gene body was lower than 1 kb upstream and downstream regions. A very low percentage of total mCs are found around transcription start and stop sites (Figure 4).

**Figure 5:**
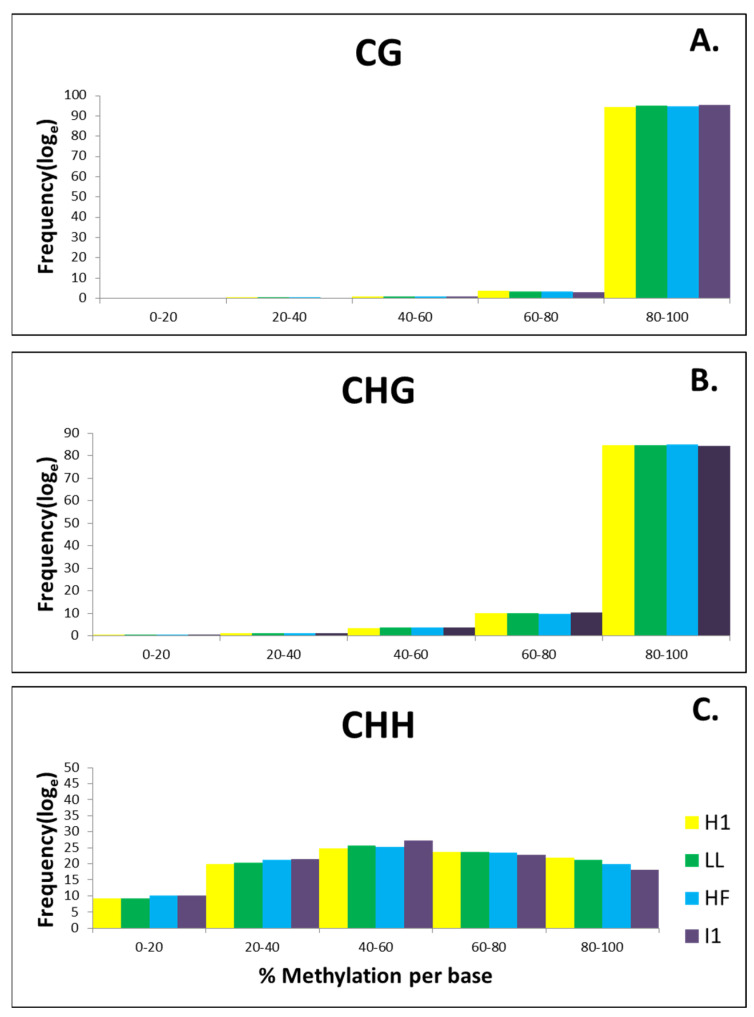
Distribution of (**A**) mCG (**B**) mCHG (**C**) mCHH level in healthy flowering (HF), phyllody-affected (I1), healthy vegetative (H1) and little leaf-affected (LL) sesame samples. The x–y axis indicates bins of percent methylation per base and frequency of total Methylcytosines in each bin, respectively.

In all four sesame samples, the majority of mCG and mCHG site methylation levels were above 90% with a unimodal distribution, whereas mCHH sites exhibited a broader distribution of methylation levels than both the mCG and mCHG sites (Figure 5A,B). Maximum locations in the CHH context have 40–60% methylation whereas approx. 20% of locations have a methylation level ranging between 80–100% (Table S3). In particular, the majority of mCHH methylation levels were between 10% and 40% (Figure 5C). 

### 3.4. DNA Methylation Patterns in Different Regions

#### 3.4.1. Distribution of Differentially Methylated Cytosines in the Genomic Regions

**Figure 6:**
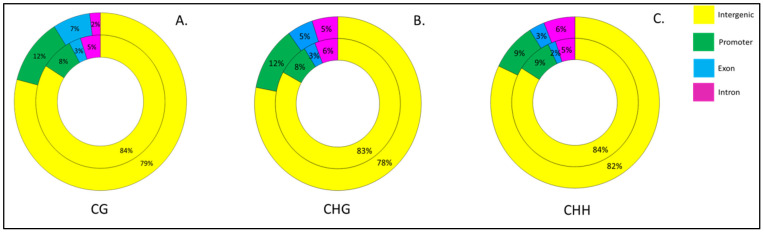
Percentage of Differentially Hypo- and Hypermethylated cytosines in promoters, exons, introns and intergenic regions in (**A**) CG (**B**) CHG and (**C**) CHH contexts. The inner circle represents phyllody (HF vs. I1) and outer circle represents little leaf affected sesame (H1 vs. LL).

Overall, our results showed that biotic stress associated with phyllody and little leaf in sesame leads to a higher percentage of methylation in intergenic regions followed by the promoter region, exonic and intronic region in CG, CHG and CHH contexts (Figure 6).

Generally, the methylation in the intergenic region was higher in little leaf (H1 vs. LL) as compared to phyllody (HF vs. I1) in the CG, CHG and CHH contexts (83–84%). The percentage of methylation in promoter and exonic region was higher in phyllody (HF vs. I1) in the CG and CHG contexts but in the CHH context the methylation % was equal in the promoter regions. The percentage of methylation in intronic regions of little leaf (H1 vs. LL) was higher than that of phyllody (HF vs. I1) in the CG and CHG context but not in the CHH context (Figure 6). 

#### 3.4.2. Identification of Differentially Methylated Cytosines (DMCs)

With a q-value < 0.01 and percent methylation difference of larger than 25%, a total of 1504 and 5744 DMCs were observed between HF vs. I1 and H1 vs. LL, respectively. Out of a total 1504 DMCs identified in HF vs. I1, 1186 were hypomethylated and only 318 DMCs were hypermethylated, whereas 3071 DMCs were hypomethylated and 2673 DMCs were hypermethylated in H1 vs. LL (Table S4).

Overall, a higher number of DMCs were found in H1 vs. LL as compared to HF vs. I1 in the CG, CHG and CHH contexts. Nearly sevenfold more DMCs were found in H1 vs. LL in the CG context (1827) compared to HF vs. I1 (251). Interestingly, the highest number of DMCs was found in the CHH context of H1 vs. LL (2268) (Table S4). 

**Figure 7:**
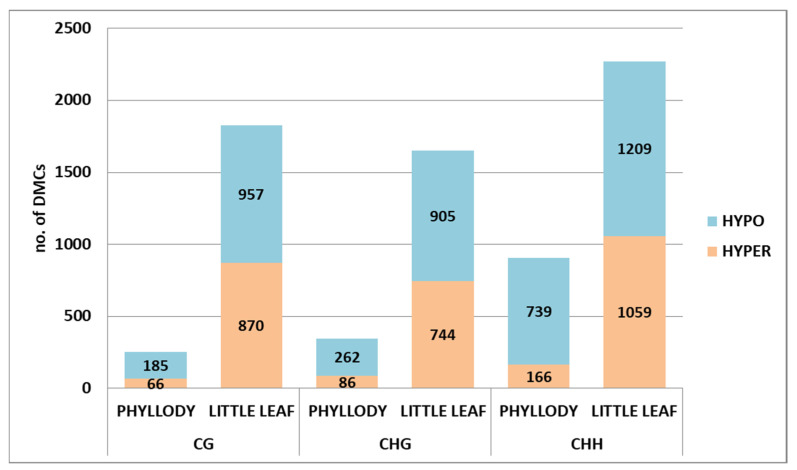
Number of Hyper- and hypomethylated Differentially Methylated Cytosines (DMCs) with percent methylation of >25% in phyllody (I1) and little leaf (LL)-affected sesame in comparison to healthy flowering (HF) and healthy vegetative (H1) sesame, respectively.

The ratio of hypomethylation vs. hypermethylation was more comparable in little leaf; however, it was much higher in phyllody-affected sesame for example, in the CHH context, DMCs were hypomethylated in HF vs. I1 (739) fivefold although an almost equal number of DMCs were hyper and hypomethylated in H1 vs. LL (1089 and 1209), respectively. These results suggest that phytoplasma induces hypomethylation to a much higher extent than hypermethylation in phytoplasma-infected sesame (Figure 7). 

#### 3.4.3. Detection of DMC-Associated Genes

To have a better understanding regarding the biological relevance of these DMCs, we identified DMCs that overlapped with genes. A total of 132 and 220 genes were identified that overlapped with differentially methylated cytosines (DMCs) in HF vs. I1 (phyllody) and H1 vs. LL (little leaf), out of which 102 genes in HF vs. I1 and 119 genes in H1 vs. LL corresponded to hypomethylated cytosines and only 30 genes overlapped with hypermethylated sites in HF vs. I1 and 101 genes overlapped with hypermethylated sites in H1 vs. LL.

**Figure 8:**
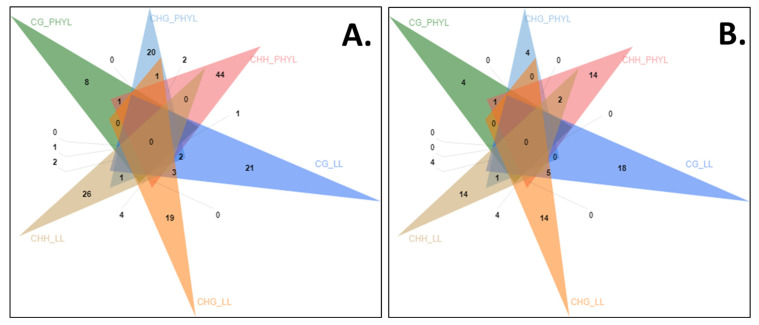
Venn diagram representing hypomethylated (**A**) and hypermethylated genes (**B**) in CG, CHG and CHH contexts. PHYL-phyllody affected sesame, LL-little leaf affected sesame.

Considering phyllody and little leaf together it was observed that in the CG context, eight genes in phyllody and 21 genes in little leaf were uniquely hypomethylated whereas four genes (~13%) in phyllody and 18 genes (~18%) in little leaf were hypermethylated. In CHH context, 44 genes (~43%) in HF vs. I1 and 26 genes (~22%) in H1 vs. LL were hypomethylated exclusively while an equal number of genes (14) were hypermethylated exclusively in both phyllody and little leaf. Almost equal numbers of genes (~20) were exclusively hypomethylated in the CHG context. However, four genes in phyllody and 14 genes in little leaf were solely hypermethylated in the CHG context (Figure 8A,B). 

When comparing the CG, CHG and CHH contexts in phyllody, two genes were hypomethylated in all three contexts (LOC105164512, LOC105168579). One gene was hypomethylated in both the CG and CHG context (LOC105162039) while LOC105178339 and LOC105179740 were hypomethylated in both the CG and CHH contexts. Similarly, LOC105179888, LOC110011415 were hypomethylated in the CHH and CHG contexts. LOC105157071, an uncharacterized locus was hypermethylated in CG and CHH context while LOC105158650, F-box protein At2g02240-like was hypermethylated in CG and CHG contexts. In little leaf seven genes were hypomethylated in CG and CHG context while four genes were hypomethylated in all the three contexts, i.e., CG, CHG and CHH contexts. Similar ratio was observed in hypermethylated genes in little leaf however, only one gene LOC105156729 was hypermethylated in all the three contexts (Figure S3).

### 3.5. Detection of Methylation in Development and Defence Related Loci

#### 3.5.1. CpG Island Prediction

Based on their vital role in defense and development of the plant, four loci were selected including two genes that were exclusively differentially methylated in little leaf affected plants (FBOX and OMT) while two were differentially methylated in both little leaf and phyllody (STP and STKL). CpG island predictions were made using MethPrimer software and it was found that FBOX locus has two CpG Islands of size 212 bp and 131 bp. Likewise two CpG islands of size 188 bp and 119 bp were found in STP locus. Only one CpG island was found in OMT locus while three CpG islands were found in STKL locus (121 bp, 102 bp and195 bp). No CpG Island was found in locus Actin and was taken as an unmethylated control for further experiments (Table 2).

**Figure 9:**
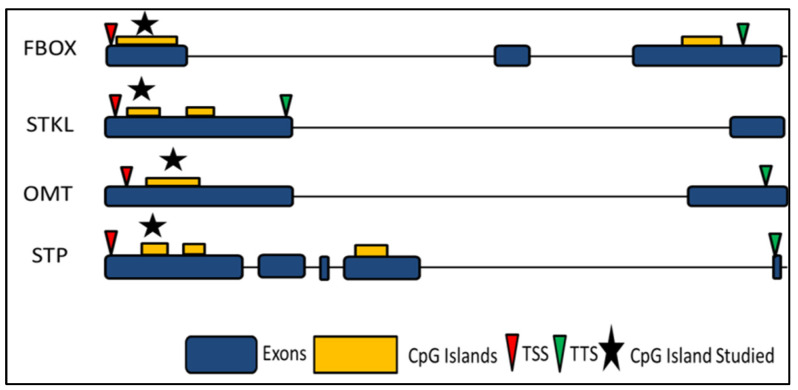
Gene structure prediction of loci namely, F-box protein PP2-B15 (FBOX), STOREKEEPER protein-like (STKL), Probable O methyltransferase 3 (OMT) and serine/threonine-protein phosphatase 7 long form homolog (STP). Blue and yellow boxes represent the exons and CpG Island, respectively. Red and green arrows indicate Transcription Start Site (TSS) and Transcription Termination Sites (TTS). Stared CpG Island has been used for methylation dependent restriction digestion and qPCR.

Gene structure predictions using GSDS 2.0 exhibited that the CpG islands in the four selected loci were present on the exonic regions. In FBOX locus, the two CpG islands fall on exon 1 and exon 3 while in STKL locus two of the CpG Islands fall on exon 1. The single CpG Island of OMT locus is present on exon 1,in case of STP locus two CpG islands are present on exon 1 while the third CpG island falls on exon 4 (Figure 9).

#### 3.5.2. Quantitative Analysis of DNA Methylation Using Real-Time PCR (qAMP)

**Figure 10:**
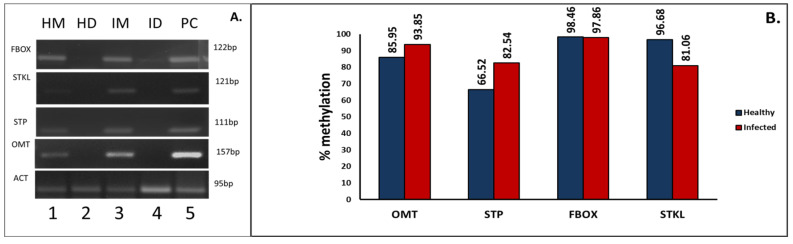
Analysis of the hypo- or hypermethylation status of selected loci namely, F-box protein PP2-B15 (FBOX), STOREKEEPER protein-like (STKL), Probable O methyltransferase 3(OMT) and serine/threonine-protein phosphatase 7 long form homolog (STP). (**A**) PCR amplicons obtained using respective primers in samples digested with McrBC enzyme for 16 h.Actin (ACT), an unmethylated control showed amplification in both mock and digested samples. HM-Healthy Mock, HD-Healthy Digested, IM-Infected Mock and ID-Infected Digested and C- Positive Control. (**B**) Graph showing percentage methylation of designated CpG Islands on the loci was calculated using ΔΔCt.

To examine whether DNA methylation is altered during phytoplasma infection, we assayed the DNA methylation levels of one CpG Island of selected loci using methylation-sensitive restriction digestion using McrBC followed by qPCR. DNA templates were digested with McrBC for 16 h and then subjected to PCR. Mock digestions were done in which the enzyme was replaced with milliQ water, these templates were called HM-healthy Mock and IM-infected Mock (Figure 10A. lane 1 and 3). Different degree of Amplification was observed in all the samples when subjected to PCR using designated primers (Figure 10A). For each set of primers a positive control (PC) was also amplified having no component of mcrBC digestion reaction (Figure 10A. lane5). Amplification in digested samples was observed using Actin primers as it was taken as unmethylated control. 

The percentage of methylation of a given site was determined using the formula % me = 100(1-e-0.7(ΔΔCt)) (Oakes et al., 2006) (Figure S4). 93.85% and 85.95% methylation of locus OMT was found in infected and healthy sample, respectively, similarly the locus STP was 82.54% methylated in infected samples while 66.52% methylated in healthy sample supporting hypermethylation upon infection. Conversely, a higher methylation percentage was observed in locus FBOX (98.46%) and STKL (96.68%) in healthy samples in comparison to infected samples, i.e., 97.86% and 81.06%, respectively, confirming hypomethylation. These results correspond with the whole-genomic bisulfite sequencing and support the alteration of DNA methylation upon phytoplasma infection (Figure 10B).

## 4. Discussion

The role of DNA methylation in controlling a variety of developmental pathways in plants was established earlier [16] However, recent reports elucidate the role of DNA methylation reprogramming during various stresses [43]. Epigenetic modification in plants as a consequence of biotic as well as abiotic stress is fast becoming an important area of investigation that is opening new avenues for understanding plant responses to environmental challenges and has vital implications in crop improvement programs aimed at developing stress-responsive plants. However, only a few such investigations have been carried out on important crop plants. Although, studies have shown that DNA methylation patterns tend to change when plants are infected with pathogens [17]. Few reports are available that elucidate the role of DNA methylation in phytoplasma infection, and it was reported that DNA methylation levels decreased in Paulownia plantlets infected with phytoplasma, suggesting the role of DNA methylation in phytoplasma infection [24]. A methylome study in phytoplasma-infected mulberry showed no significant difference in the average methylation level of infected leaves when compared to healthy leaves, there were 1253 differentially methylated genes (DMGs) and 1168 differentially expressed genes (DEGs) in the infected leaves, and 51 genes were found simultaneously to be differently methylated and expressed [22]. Studies are completely lacking on the genome-wide methylome profiling of phytoplasma-affected sesame plants vis-a-vis asymptomatic plants. Here, we report the genome-wide DNA methylation study in sesame for the first time, indicating the alteration of DNA methylation patterns upon phytoplasma infection. 

### 4.1. Global DNA Methylation Profile

In our study, the overall absolute methylation percentage ranged from 40–46%. The relative methylation percentage that takes the sequence length under consideration was found to be two fold higher in healthy flowering when compared to phyllody-infected plants; however, the difference was negligible in little leaf vs. healthy vegetative plant. Thus, responses in the overall methylation level change depending on the stage at which infection occurred and consequently on the symptoms developed upon infection.

In CG and CHG contexts, a marked increase in the fraction of mCs was observed in phyllody-affected plants; on the contrary, the percentage of mCs decreased in the CHH context in phyllody-affected plants. The same pattern was observed in little leaf vs. healthy vegetative samples but the difference was not particularly significant (Figure 2).

On comparing the proportion of CG, CHG and CHH methylation in different plants, a varied pattern was observed. In *Sesamum indicum*, the methylation proportion in various contexts closely matches with that in *Glycine max* (mCG 35.088%, mCHG 33.95% and mCHH 30.962%), which is also an oilseed crop [44]. *Nelumbo nucifera* also follows a similar pattern (mCG 31.6%, mCHG 34.4%, mCHH 34.0%) [45]. In *Arabidopsis thaliana*, the major proportion (41.6%) of mCs was found in the CG context, followed by the CHH (36.5%) and CHG (21.6%) contexts [46]. In *Vigna radiata*, CHH context methylation occupies the highest proportion (54.34%) followed by CHG (24.57%) and CG (21.09%) methylation [47]. A lack of correlation between the size of the genome, GC content, chromatin structure and global methylation level fail to clearly comprehend such a huge variation in DNA methylation profiles among different plant species.

The percentage of cytosines methylated over total cytosines in each sequence context (CG, CHG and CHH) also shows a wide variation among different plant species. In healthy flowering *Sesamum indicum* L. (H1), ~85% of the total CGs, ~74% of total CHGs and ~26% of total CHHs were methylated and a similar trend was observed in healthy vegetative and little leaf-affected sesame. In case of phyllody infection (I1), percentage of CG methylation (~85%) is similar as in healthy sample. A slight reduction (73.81% to 71.52%) in CHG-context methylation occurs, whereas an extensive reduction (26.26% to 18.12%) occurred in CHH-context methylation upon infection suggesting a probable role of hypomethylation in the CHH context in activating or silencing genes in response to phytoplasma infection (Figure 3). It was observed that sesame has comparatively higher ratios of context-specific methylation as compared to other plant species. For example, in *Glycine max*, 51% of total CGs are methylated, which is much lower than that in sesame (~85%), [44]. The methylation ratios in the CG context in different plant species are as follows: *Arabidopsis* thaliana (22.26%), *Oryza sativa* (59.39%), *Populus trichocarpa* (41.87%), *Vigna radiata* (58.9%), *Betula* (27.43%), *Nelumbo nucifera* (58.4%) and *Brassica rapa* (36.5%) [45,46,47,48,49]. The CG context methylation ratio of *Sesamum indicum* closely matches with that of *Zea mays* (86%) [50].

Similarly, the CHG context methylation ratio in sesame (69–74%) matches with that of maize (74%). *Arabidopsis* has a very low (5.92%) ratio of CHG context methylation. *Oryza sativa* and *Populus trichocarpa* have similar CHG methylation ratios (~21%). *Vigna radiata*, *Gycine max*, *Betula platyphylla*, *Nelumbo nucifera* and *Brassica rapa* have CHG methylation ratios of 51.5%, 39%, 23.71%, 40.6% and 13.4%, respectively [44,45,46,47,48,49].

The CHH context methylation ratio in healthy and phytoplasma-infected sesame shows a considerable difference in phyllody symptom development but not in little leaf although the range remains the same, i.e., 18–20%. The CHH methylation ratio of sesame is comparable with that of *Vigna radiata* (17.9%). *Arabidopsis thaliana*, *Oryza sativa*, *Populus trichocarpa*, *Zea mays*, *Nelumbo nucifera, Brassica rapa* have low CHH-context methylation ratios of 1.51%, 2.18%, 3.25%, 5.4%7.5% and 5.3%, respectively. Strikingly, the CHH-context methylation in *Glycine max* is very low (0.05%). Therefore, of the nine taxa for which the detailed methylome studies were conducted and which were compared, eight taxa exhibited the lowest methylation ratio in CHH as compared to CG- and CHH-methylation ratios, and a similar trend was observed in sesame. *Betula platyphylla* is an exception to this trend, with the highest methylation ratio (48.86%) observed in the CHH context [48].

### 4.2. Degree of Methylation in Different Contexts in Healthy and Infected Samples

In our study, it was observed that methylation in the CHH context is more dynamic and the proportion of methylated cytosines o 80–100% methylation decreases upon infection, indicative of asymmetric CHH-context hypomethylation in phytoplasma-infected sesame. On the other hand, the percentage of sites with a 80- 100% methylation level is almost similar in healthy and phytoplasma-infected samples in both CG (~95%) and CHG contexts (~85%) (Figure 5).

Cokus et al. [51] reported that most of the CGs in *Arabidopsis thaliana* have either a very high (80–100%) methylation level or are unmethylated. CHG sites have a uniform distribution with methylation levels varying from 20% to 100% and CHH sites have very low methylation levels varying from 0% to 10%. Zemach et al. [52] also reported similar levels of CG and CHG methylation in different organs (embryos, shoots, roots and leaves) in rice, whereas the CHH-context methylation level showed dynamism with increasing levels of methylation from the embryo stage to young shoot, with the maximum methylation level found in mature leaves of rice. These findings suggest that methylation levels dynamically change in accordance with the stage of the plant as well as external environmental conditions.

### 4.3. Differentially Methylated Cytosines (DMCs)

The RNA-directed DNA methylation (RdDM) pathway is mainly involved in establishing DNA methylation in all three contexts. However, methylation in different sequence contexts is maintained by distinct pathways [53]. Methylation at the CG context is maintained by METHYLTRANSFERASE 1 (MET1) with the involvement of Ubiquitin-like with PHD and RING Finger domain 1 factor to target hemimethylated sites. Plant-specific chromomethylases such as CMT3 in *Arabidopsis thaliana*, which is involved in the maintenance of CHG methylation, are also responsible for the di-methylation of H3K9 [54]. Lastly, the maintenance of CHH methylation is performed either by the RdDM pathway or CMT2 pathway [55]. In this study, the highest number of differentially methylated cytosines (DMCs) was observed in the CHH context followed by the CHG and CG contexts. It is important to point out that the RNA-directed DNA methylation 4-like (*RDM4*) gene has been found to be differentially methylated in the phyllody-affected plant suggesting its role in the drastic differences in the CHH-context methylation observed. The *RDM4* gene encodes a highly conserved transcription factor which has been found to be involved in the transcriptional regulation of RNA Pol V and Pol II and thus, is essential for RdDM [56]. Loss-of-function of *RDM4* leads to the impairment of the RdDM pathway by affecting Pol V, which is known to be involved in the transcription of intergenic regions [57].

Further, there are four different enzymes responsible for the maintenance of methylation in *A. thaliana*, namely, DNA METHYL TRANSFERASE (MET1), CHROMOMETHYLASE (CMT3), DOMAIN REARRANGED METHYL TRANSFERASE 2 (DRM2) and CHROMOMETHYLASE (CMT2), but the establishment of methylation is attributed exclusively to an enzyme in the RNA-directed DNA methylation (RdDM) pathway viz. enzyme DRM2 [58]. Using co-expression data to identify genes that are closely regulated and coregulated in the RdDM pathway, [58] two putatively chromatin-modifying proteins belonging to the SNF2 family of helicase-like protein were identified, namely SNF2-RING-HELICASE-LIKE-1 and 2 (FRG1 and 2). A phylogenetic analysis of SNF2 superfamily demonstrated its closeness to RING type E3 ligase that has a role in the ubiquitylation of small ubiquitin-like modifier conjugated substrates. We recently identified an ortholog of SAP54 in phytoplasma-infected sesame [59]. Interestingly, the RING-type E3 ligase was identified as one of the strongest interacting partners of the effector molecule S54LP (ortholog of SAP54 from phytoplasma affected sesame) in our Y2H studies (data unpublished). Further, Sridhar et al. [60] previously demonstrated that non-CpG DNA methylation is actively mediated by Ubiquitin protease UBP26. In fact, the process leading to CHH methylation by the RdDM pathway involving Ubiquitin protease (UBP26) has been reported to be closely linked with histone H2B de-ubiquitylation. Therefore, we hereby propose that a significant difference between the levels of CHH methylation in healthy and phytoplasma-infected sesame plants seemingly involves modulating the ubiquitination levels of crucial components mediated by the interaction between phytoplasma secreted effector molecule SAP54 and the host plant E3 ubiquitin ligase.

The demethylation of genes related to UPS (ubiquitin/proteasome system), namely MATH-BTB domain protein (a proteasome component domain protein) and ubiquitin protein ligase, was observed in roots of soybean in response to soybean cyst nematode infection, leading to the overexpression of these genes in the infected roots [61]. Thus, differential methylation has been proposed to be an accessory mechanism targeting host UPS and other cellular processes in addition to dysregulation caused by the pathogen effectors.

In our study, few genes were found to be differentially methylated (hypo- or hyper) in more than one context. This further substantiates the involvement of different enzymes in mediating methylation in the three contexts.

### 4.4. Hypomethylation More Pronounced than Hypermethylation in Phyllody Symptom Development

Our study revealed that Phytoplasma infection leading to phyllody symptoms results in a significantly higher degree of hypomethylation (79%) as compared to hypermethylation. Further, among the hypomethylated DMCs, 74–75% of DMCs were hypomethylated in the CG and CHG contexts, whereas in the CHH context, hypomethylation was more pronounced and ~82% of the total DMCs were hypomethylated. Interestingly, the hyper and hypomethylated DMCs in all the contexts were almost equal in little leaf-affected sesame (Table S4). 

Dynamic methylation changes in *Arabidopsis* were observed upon infection with *Pseudomonas syringae* pv *tomato* DC3000 (*Pst*) in [20]. It was reported that hypomethylation in differentially methylated regions in response to infection by *Pst* induced the expression of defense-responsive genes in *Arabidopsis thaliana.* Yu et al. [21] also delineated the role of DNA demethylation in activating defense-related genes and transposable elements in response to infection by *Pst* in *Arabidopsis*. In fact, they also revealed the role of DNA methylation in prohibiting the proliferation and vascular propagation of the bacteria in *Arabidopsis* leaves. Rambani et al. [61] revealed the induction of DNA hypomethylation to a larger extent as compared to hypermethylation in soybean upon infection with soybean cyst nematode, thus leading to a larger number of SCN-responsive genes being up-regulated than down-regulated. Satge et al. [62] reported that the demethylase gene *DEMETER* (DME) is strongly expressed during nodule development in *Medicago truncatula*, thus resulting in the reprogramming of DNA methylation which leads to the upregulation of genes, especially nodule-specific cysteine-rich (NCR) genes. In our case, hypomethylation was more pronounced in phyllody as compared to little leaf. 

### 4.5. Prevalence of Gene Body Methylation (gbM) upon Phytoplasma Infection

The location, the DNA sequence and the context of cytosine methylation determine the functional consequences of DNA methylation [63]. The degree of methylation, the methylation context and its distribution within the gene provide insight into the enzymatic pathway of methylation, the ontology of the gene, gene expression level, size of the gene and the rate of nucleotide substitutions [64,65].

Changes in DNA methylation can modify gene transcription levels under particular conditions, including abiotic and biotic stresses, and lead to observable phenotypes. Many studies demonstrated that DNA methylation plays a significant role in the control of gene expression in plants such as dandelions, maize, Arabidopsis, and *Pyropia haitanensis* in response to various abiotic and biotic stimuli [66,67,68,69]. When apples were subjected to water stress, varied expression levels of methylation-related genes encoding DNA methyltransferases and demethylases were observed [70]. The mRNA level of some methyltransferase and demethylase genes also had significant differences under drought stress in mulberry [71].

In this study, the differentially methylated genes (DMGs) in all the three sequence contexts exhibited either hypo- or hypermethylation in the gene bodies, thus highlighting the importance of investigating the effects of gene body methylation or demethylation on gene expression and providing a future line of investigation for related work. The occurrence of non CpG hypo- or hypermethylation in the gene body was observed in soybean in response to infection with cyst-nematode [61]. However, earlier studies reported that gene body methylation is mainly restricted to the CG context [72]. The possibility of biotrophic interaction inducing non-CpG gene body methylation in plants is made evident by this study. 

The extent of gene body methylation determines the expression level of the gene. Moderately methylated gene bodies have been reported to promote the increased expression of genes. On the other hand, an inhibition of the expression of genes with low or high methylated bodies was observed [73,74,75,76].

The gene body regions of plants are frequently highly methylated, but the transcriptional start sites (TSS) and transcriptional termination sites (TTS) are rarely methylated. In addition, numerous intergenic regions are hypermethylated, whereas most promoter regions are hypomethylated [73]. DNA methylation was enriched in intergenic and gene body areas after phytoplasma infection in mulberry, although promoter regions were hypomethylated. Furthermore, the methylation level in exons was found to be higher than that in introns [22]. These results indicated that the DNA methylation profiles of sesame are analogous to that of mulberry, and the methylation patterns among different plant species may be conservative. Epigenetic markers are known to transmit acquired environmentally adaptive traits to subsequent generations. Several examples of DNA methylation pattern heritability have been described in different plant species [77]. Phenotypic diversity and geographic expansion were found to be linked to epigenetic modifications during the domestication of cotton [78]. The intergenerational transmission of phenotypic traits such as roots adapted to drought stress, a delayed flowering time and modified plant architecture caused by DNA methylation were also reported [79,80]. Because of its heritable nature, the removal of DNA methylation marks has been a focus of research to remodel the expression of genes already present in a genome. 

An association between gene body methylation (gbM) and transcription was observed. Methylation in the vicinity of the transcription start site has been reported to block initiation, but gene body methylation has no role in hindering transcription initiation. Moreover, gene body methylation also plays a role in stimulating transcription elongation [81]. The location of gbM within the coding region and its effects on phenotypes are suggestive of its crucial roles in regulating splicing and gene expression [82]. The possible role of mCG in inhibiting RNA polymerase II and the initiation of transcription explain its depletion at transcription start sites. Moreover, genes with mCG at TSS have low expression levels.

In humans, it has been reported that gene body methylation is often associated with active transcription [83]. This phenomenon is referred to as the ‘DNA methylation paradox’ [84]. Extensive studies confirming a positive correlation between gene body methylation and active transcription on the active X-chromosome have been conducted [85]. Shotgun bisulphite sequencing of the genomes of plants and animals also suggests a positive correlation between gbM and transcription [46,86]. Jones et al. [81] stressed the positive correlation between gbM and transcription elongation in spite of the fact that the CpG methylated sites in the gene body are marked by H3K9me3 and thus, these sites are bound by methyl-CpG-binding protein 2 (MECP2), which is known to suppress transcription when located at the transcription start site [87]. This correlation makes relevant the ‘DNA methylation paradox’ which suggests that methylation in the upstream region of the gene body leads to gene silencing or reduced gene expression, whereas gene body methylation has a positive correlation with gene expression [84]. Thus, the mere presence of a methylation mark is not indicative of its role in regulating transcription. The genomic and cellular context of the methylated site is crucial in explicating its impact on the regulation of transcription [81].

### 4.6. Intergenic Regions Show Highest Proportion of Differentially Methylated Cytosines

In the present study, the highest proportion (78–82% in different contexts) of differential methylation was observed in intergenic regions as compared to promoter, exonic and intronic regions. In maize, the degenerate relics of retrotransposons in the intergenic regions contribute to higher degree of methylation in this region [88]. The analysis of human chromosomes 21 and 22 also showed high proportions of CpG islands in the intergenic regions which are targeted for methylation [89]. However, the functional significance of CpG islands in the intergenic regions remains elusive.

### 4.7. Development and Defense-Related Genes Are Methylated in Response to Phytoplasma in Sesame

DNA cytosine methylation and demethylation events both occur in different plants after pathogen infection [18,90], although evidence indicates that these changes are species and organ-specific [91]. Previous studies have shown the reversion of flowering related genes upon phytoplasma infection and phyllody symptom development [8,9]. Keeping this in mind, an attempt was made to explore the cytosine methylation status of selected key flowering regulatory genes, namely UNUSUAL FLORAL ORGANS (UFO), FLORICAULA/LFY (FLO), FLOWERING LOCUS T- like (FLT) and SUPERMAN (SUP). However, DNA methylation using the mcrBC assay was not detected in these key flowering regulator genes [92]. Recently, we successfully detected the presence of CpG Islands in seven defense and development-related loci [34]. Therefore, we further explored the methylation status of four out of these seven loci in the present study. The McrBC assay could also successfully detect cytosine methylation in loci, namely F-box protein PP2-B15 (FBOX), STOREKEEPER-protein-like (STKL), Probable O-methyl- transferase 3 (OMT) and Serine/threonine-protein phosphatase 7 long form homolog (STP). Further, loci FBOX and STKL were found to be hypomethylated and loci OMT and STP were detected as being hypermethylated by WGBS and mcrBC-qPCR.

P-proteins are structural proteins that close the pores in the sieve plate to seal off damaged sieve components. The deposition of callose and P-proteins in the sieve pores of the leaf phloem’s sieve elements was reported during the symptomatic stage of phytoplasma infection [93]. The hypomethylation of F-box protein PP2-B15 (FBOX) locus, a P-protein observed in the present study, suggests the role of DNA methylation in the deposition of callose and P-proteins upon phytoplasma infection. 

A locus STP was found to be hypermethylated upon phytoplasma infection, widely known as Protein phosphatase 7(PP7). Under white and blue light, it works as a positive regulator of cryptochrome signaling, which is involved in hypocotyl growth inhibition and cotyledon enlargement [42]. Protein phosphatase 2A (PP2A), a homolog of STP or PP7, is a multifunctional serine/threonine phosphatase that is made up of the following three subunits: a catalytic C, a structural A, and a regulatory B. The key to the functional specificity and control of PP2A is the assembly of the complex with the proper B subunit. According to new data, the methylation and phosphorylation of the PP2A C subunit play a critical part in this process. PP2 genes have also been known to play an important role in plant biotic stresses [94]. 

According to Zourelidou et al. [40] mutations in a conserved region of the patatin promoter’s B-box lower the tuber-specific and sucrose-inducible gene production of class I patatin in transgenic potato plants. Storekeeper (STK), a DNA binding protein recognizes the B-box motif and regulates patatin expression in potato tubers via the B-box motif. In tobacco, the ectopic expression of *Arabidopsis thaliana stk01* and *stk03* enhanced the number of vegetative internodes, leaf size, stem diameter, and sturdiness. The production of rosettes was the first step in the development of these plants, which was followed by a delay in shoot elongation and flowering. Yeast Two-Hybrid screens also revealed that STK01 and STK03 can form homodimers and heterodimers with other STK-related proteins [39]. The hypomethylation of such an important gene upon phytoplasma infection suggests its role in causing similar exaggerated symptoms in sesame.

O-methyltransferases (OMTs) are a class of enzymes that transfer a methyl group from S-adenosyl-L-methionine to its acceptor substrates. OMTs are classified into different classes based on their structural characteristics [95]. These enzymes are known to methylate the oxygen atom in phenylpropanoids, flavonoids, and alkaloids, among other secondary metabolites. In plants, O-Methylation is important for lignin production, stress tolerance, and disease resistance [41]. They are engaged in phenolic and flavonoid processes in *Gossypium* species. Phenolics protect the cellulose fiber from harmful external biotic and abiotic stressors, boosting plant cell wall strength and growth [95].

The lignin biosynthetic route has been linked to plant cafeic acid 3-O-methyltransferase (COMT), which catalyses the methylation processes of hydroxylated monomeric lignin precursors, which are then involved in lignin production. In response to *Rhizoctonia cerealis* infection, the wheat COMT gene TaCOMT-3D was discovered. Sharp eyespot susceptibility has increased in TaCOMT-3D-silenced wheat plants, although overexpression improved sharp eyespot resistance, stem mechanical strength, and lignin buildup in transgenic wheat lines [11]. The hypermethylation of the OMT gene was hereby reported in our study. Since the gene expression pattern could be affected by changes in DNA methylation levels, the hypermethylation of OMT upon phytoplasma infection might alter lignin biosynthesis in infected sesame plants. It is important to point out that the stem sturdiness reported in phytoplasma infection is also evident in sesame [96].

## 5. Conclusions

To the best of our knowledge, this work provides the first high resolution genome-wide DNA methylation maps of healthy and phytoplasma-infected sesame genome. Given the important role of DNA methylation in phytoplasma infection and control of gene expression, these data should serve as an important genomic resource for the scientific community.

The pattern of sesame methylation is similar to most other plant species and the percentage of cytosines methylated over total cytosines was highest in CG, CHG and lowest in CHH in healthy and phytoplasma-infected sesame. An extensive reduction in methylation in the CHH context suggests a putative role of CHH hypomethylation in gene expression during phyllody symptom development. Further, a higher number of DMCs were hypomethylated in the CHH context in phyllody-affected sesame, although the number of hypo or hypermethylated DMCs was equal in little leaf-affected sesame, suggesting the involvement of different mechanisms.

DMCs in all three sequence contexts demonstrated either hypo or hypermethylation in the gene body, but intergenic regions showed higher differential methylation as compared to promoter, exonic and intronic regions in both phyllody and little leaf-affected sesame.

The methylation status of four development and defence-related loci was confirmed using a quantitative analysis of DNA methylation using real-time PCR (qAMP) and it was found that loci STOREKEEPER protein-like and F-box protein PP2-B15, were hypomethylated. Likewise, serine/threonine-protein phosphatase-7 homolog and probable O-methyltransferase 3 loci were detected to be hypermethylated.

Overall, the comprehensive investigation of differential methylation in different genomic regions in all the three contexts in phytoplasma-infected sesame plants provides vital information on the molecular mechanism underlying symptom development that can prove useful in programs aimed at developing phytoplasma resistance in sesame. Future studies will be focused on exploring the expression and function of these DNA-methylated genes. 

## Figures and Tables

**Table 1:** Summary of genome-wide methylation sequencing data. HF-healthy Flowering, I1-Phyllody, H1-healthy vegetative and LL-little leaf sesame samples.

Terms	HF	I1	H1	LL
Raw reads number	42,438,902	25,751,266	48,702,274	37,065,786
Clean reads number	42,066,562	25,205,760	47,335,434	36,714,884
Clean reads rate (%)	99.12	97.88	97.19	99.05
Uniquely mapped reads	34,568,665	21,403,088	39,974,252	31,246,777
Uniquely mapped rate (%)	82.17	84.91	84.44	85.10
GC%	35	34	33	33

**Table 2:** List of selected loci used for methylation dependent restriction digestion and qPCR.

Loci	Name	Methylation Status Based on WGBS	Length (bp)	No of Exons	CpG Island Length (bp)	CpG Island Position	Function
LOC105172401	F-box protein PP2-B15(FBOX)	Hypo	2512	3	Island 1–212	(48–259)	Plugging of sieve plates to maintain turgor pressure in sieve tubes after sieve element injury is the most commonly accepted role for these proteins [38].
					Island 2–131	(2125–2255)	
LOC105159820	STOREKEEPER protein-like (STKL)	Hypo	3859	2	Island 1–188	(188–375)	The STOREKEEPER (STK) family of DNA-binding proteins functions as transcription factors. Ectopic expression of two Arabidopsis thaliana stk-like genes, *stk01* (At1g61730) and *stk03* (At4g00238), in tobacco increases the number of vegetative internodes and preferred plant and leaf size, stem diameter and hardiness [39]. It is also known to regulate patatin expression in potato tubers through the B-box motif [40].
					Island 2–119	(567–685)	
LOC105171976	Probable O methyltransferase 3 (OMT)	Hyper	3016	2	Island 1–240	(189–428)	Plant O-methyltransferases (OMTs) are a broad family of enzymes that methylate the oxygen atom in phenylpropanoids, flavonoids, and alkaloids. In plants, O-Methylation is important for lignin production, stress tolerance, and disease resistance [41].
LOC110012292	serine/threonine-protein phosphatase 7 long form homolog (STP)	Hyper	3801	5	Island 1–121	(239–359)	Under white and blue light, STP seems to operate as a positive regulator of cryptochrome signaling, therefore involved in hypocotyl development inhibition and cotyledon expansion [42].
					Island 2–102	(475–576)	
					Island 3–195	(1360–1554)	
LOC105161467	Actin (ACT)	-	3865	5		-	Unmethylated control

## Data Availability

All new research data were presented in this contribution.

## Supplementary Materials

The following supporting information can be downloaded at: https://www.mdpi.com/article/10.3390/biology11070954/s1, Figure S1: Scatter plots of percentage methylation values representing correlation between Phyllody (HF vs. I1) (a–c) and Little Leaf (H1 vs. LL) (d–f). Figure S2: Read coverage per base in Healthy Flowering (HF), Phyllody affected (I1), Healthy vegetative (H1) and Little Leaf (LL) affected sesame samples in CG context (A–D), CHG context (E–H) and CHH context (I–L). Figure S3: Venn diagram representing hypomethylated genes (A, B) and hypermethylated genes in PHYL and LL (C, D). PHYL-phyllody affected sesame, LL-little leaf affected sesame. Figure S4: ΔΔCT values of four loci namely, Probable O methyltransferase 3 (OMT), serine/threonine-protein phosphatase 7 long form homolog (STP), FBOX-PP2-B15 (FBOX) and STOREKEEPER protein-like (STKL) in healthy and phytoplasma-infected samples. Actin was used as endogenous control. Each point represents the mean value of three replications ± SD. Table S1. Correlation coefficient between Phyllody (HF vs. I1) and Little Leaf (H1 vs. LL) affected sesame. Table S2. Absolute and relative methylation percentage in HF-Healthy Flowering, I1-Phyllody, H1-Healthy vegetative and LL-Little Leaf sesame samples. Table S3. Methylation level (%) of cytosines in different (CG, CHG and CHH) sequence contexts in HF-Healthy Flowering, I1-Phyllody, H1-Healthy vegetative and LL-Little Leaf sesame samples. Table S4. Hyper and hypomethylated DMCs with methylation difference of more than 25% in different sequence contexts in Phyllody (HF vs. I1) and Little Leaf (H1 vs. LL) affected sesame.
