# Supplementary material for: Comparative DNA Methylome of Phytoplasma Associated Retrograde Metamorphosis in Sesame (Sesamum indicum L.)
Source: Biology (Basel). 2022 Jun 23;11(7):954. doi: 10.3390/biology11070954 (PMC9311523; doi:10.3390/biology11070954)
Supplement: Supplementary file 1 [file biology-11-00954-s001.zip › supplementary figures Biology special issue phytoplasma.pdf]

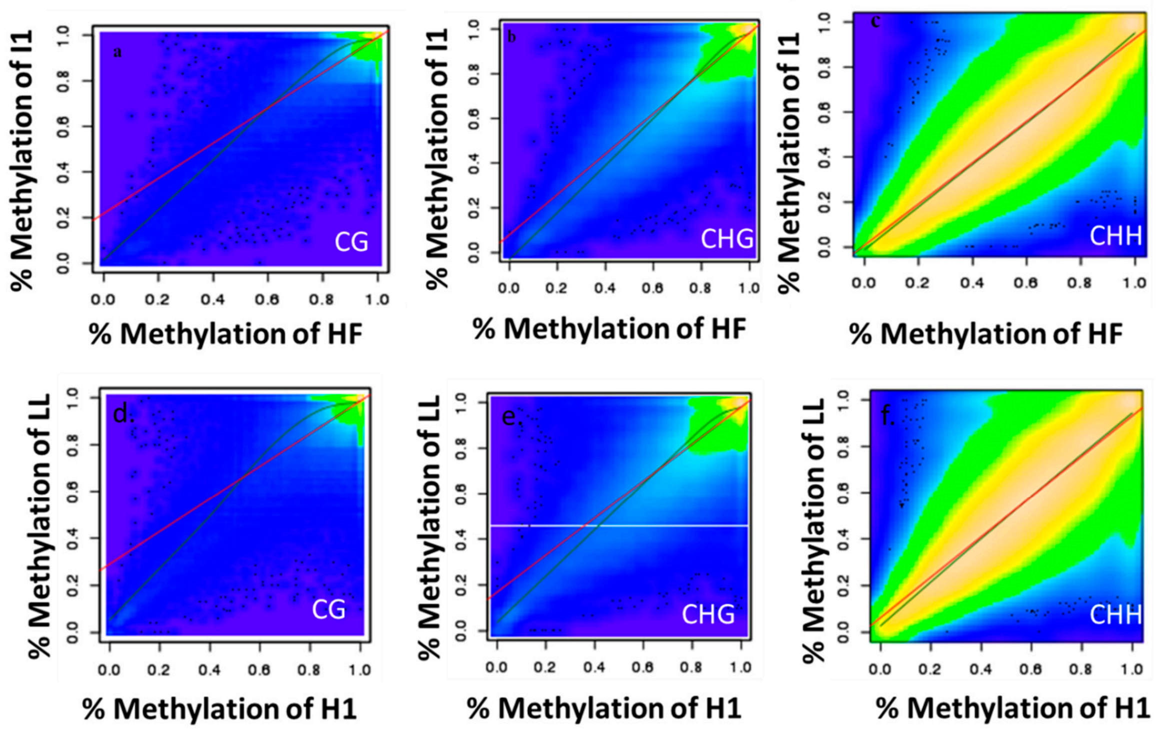

Figure S1: Scatter plots of percentage methylation values representing correlation between Phyllody (HF vs I1) (a, b and c) and Little Leaf (H1 vs LL) (d, e and f).

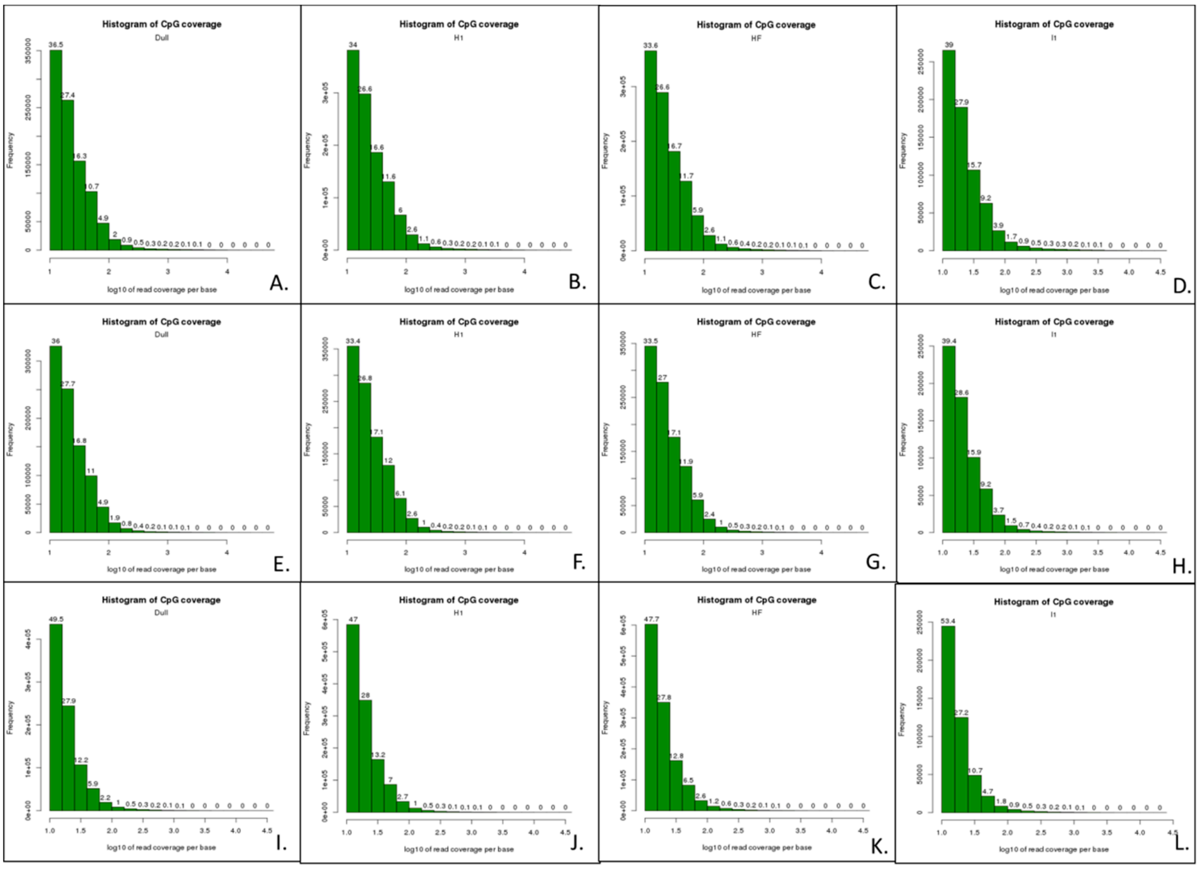

Figure S2: Read coverage per base in Healthy Flowering (HF), Phyllody affected (I1), Healthy vegetative (H1) and Little Leaf (LL) affected sesame samples in CG context (**A**, **B,** **C** and **D**), CHG context (**E**, **F, G** and **H**) and CHH context (**I**, **J, K** and **L**).

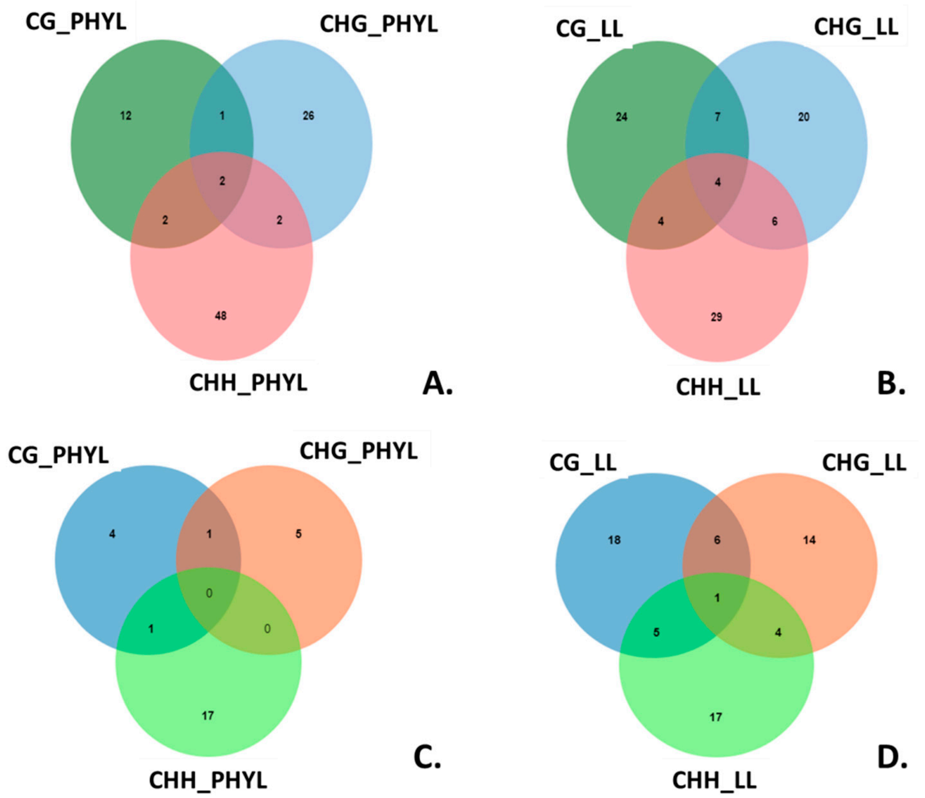

Figure S3: Venn diagram representing hypomethylated genes (**A** & **B**) and hypermethylated genes in PHYL and LL(**C** & **D**). PHYL-phyllody affected sesame, LL-little leaf affected sesame.

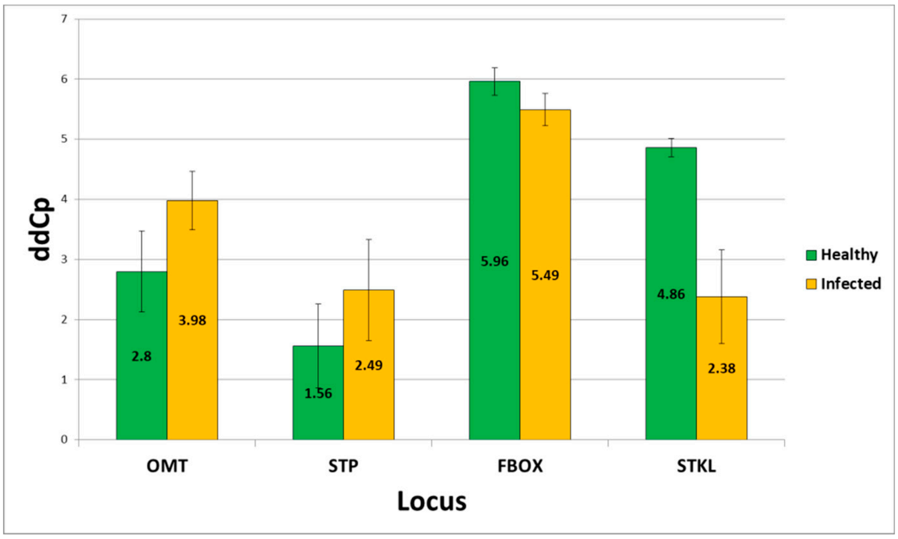

Figure S4: ΔΔCT values of four loci namely,Probable O methyltransferase 3(OMT), serine/threonine-protein phosphatase 7 long form homolog(STP), FBOX-PP2-B15(FBOX) and STOREKEEPER protein-like(STKL) in healthy and phytoplasma infected samples. Actin was taken as endogenous control.Each point represents the mean value of three replications ± SD.
