# Supplementary material for: Comparative DNA Methylome of Phytoplasma Associated Retrograde Metamorphosis in Sesame (Sesamum indicum L.)
Source: Biology (Basel). 2022 Jun 23;11(7):954. doi: 10.3390/biology11070954 (PMC9311523; doi:10.3390/biology11070954)
Supplement: Supplementary file 1 [file biology-11-00954-s001.zip › supplementary tables Biology special issue phytoplasma.pdf]

Table S1. Correlation coefficient between Phyllody (HF vs I1) and Little Leaf (H1 vs LL) affected sesame.

**Context**	**Correlation coefficient**	
	HF vs I1	H1 vs LL
CG	0.76	0.71
CHG	0.83	0.79
CHH	0.87	0.86

Table S2. Absolute and relative methylation percentage in HF-Healthy Flowering, I1-Phyllody, H1-Healthy vegetative and LL-Little Leaf sesame samples.

**Sample**	**Absolute Methylation percentage (%)**	**Relative Methylation percentage (%)**
**HF**	46.20	66.32
**I1**	41.39	35.11
**H1**	41.06	64.09
**LL**	40.52	52.55

Table S3. Methylation level (%) of cytosines in different (CG, CHG and CHH) sequence contexts in HF-Healthy Flowering, I1-Phyllody, H1-Healthy vegetative and LL-Little Leaf sesame samples.

**Methylation level (%)**	**Percentage (%) of methylated cytosines**											
	**CG**				**CHG**				**CHH**			
	**H1**	**LL**	**HF**	**I1**	**H1**	**LL**	**HF**	**I1**	**H1**	**LL**	**HF**	**I1**
0-20	0.4	0.4	0.3	0.3	0.6	0.5	0.4	0.5	9.3	9.1	10.1	10.2
20-40	0.5	0.5	0.5	0.4	1.3	1.3	1.3	1.1	19.9	20.3	21.2	21.5
40-60	0.9	0.8	1.0	0.8	3.5	3.6	3.6	3.7	24.9	25.6	25.3	27.2
60-80	3.6	3.4	3.3	3.0	9.9	10	9.8	10.4	23.7	23.8	23.4	22.9
80-100	94.4	95	94.8	95.4	84.7	84.7	85.1	84.3	21.9	21.3	20.0	18.2

Table S4. Hyper and hypomethylated DMCs with methylation deference of more than 25% in different sequence contexts in Phyllody (HF vs I1) and Little Leaf (H1 vs LL) affected sesame.

**Context**	**Filtered DMCs**	**Hyper methylated DMCs**		**Hypo methylated DMCs**	
		Number of DMCs	Percentage	Number of DMCs	Percentage
HF vs I1_CG	251	66	26.29	185	73.71
HF vs I1_CHG	348	86	24.71	262	75.29
HF vs I1_CHH	905	166	18.34	739	81.66
TOTAL	1504	318	21.14	1186	78.86
H1 vs LL_CG	1827	870	47.62	957	52.38
H1 vs LL_CHG	1649	744	45.12	905	54.88
H1 vs LL_CHH	2268	1059	46.69	1209	53.31
TOTAL	5744	2673	46.54	3071	53.46
