# Supplementary figures and images for: Comparative DNA Methylome of Phytoplasma Associated Retrograde Metamorphosis in Sesame (Sesamum indicum L.)
Source: Biology (Basel). 2022 Jun 23;11(7):954. doi: 10.3390/biology11070954 (PMC9311523; doi:10.3390/biology11070954)

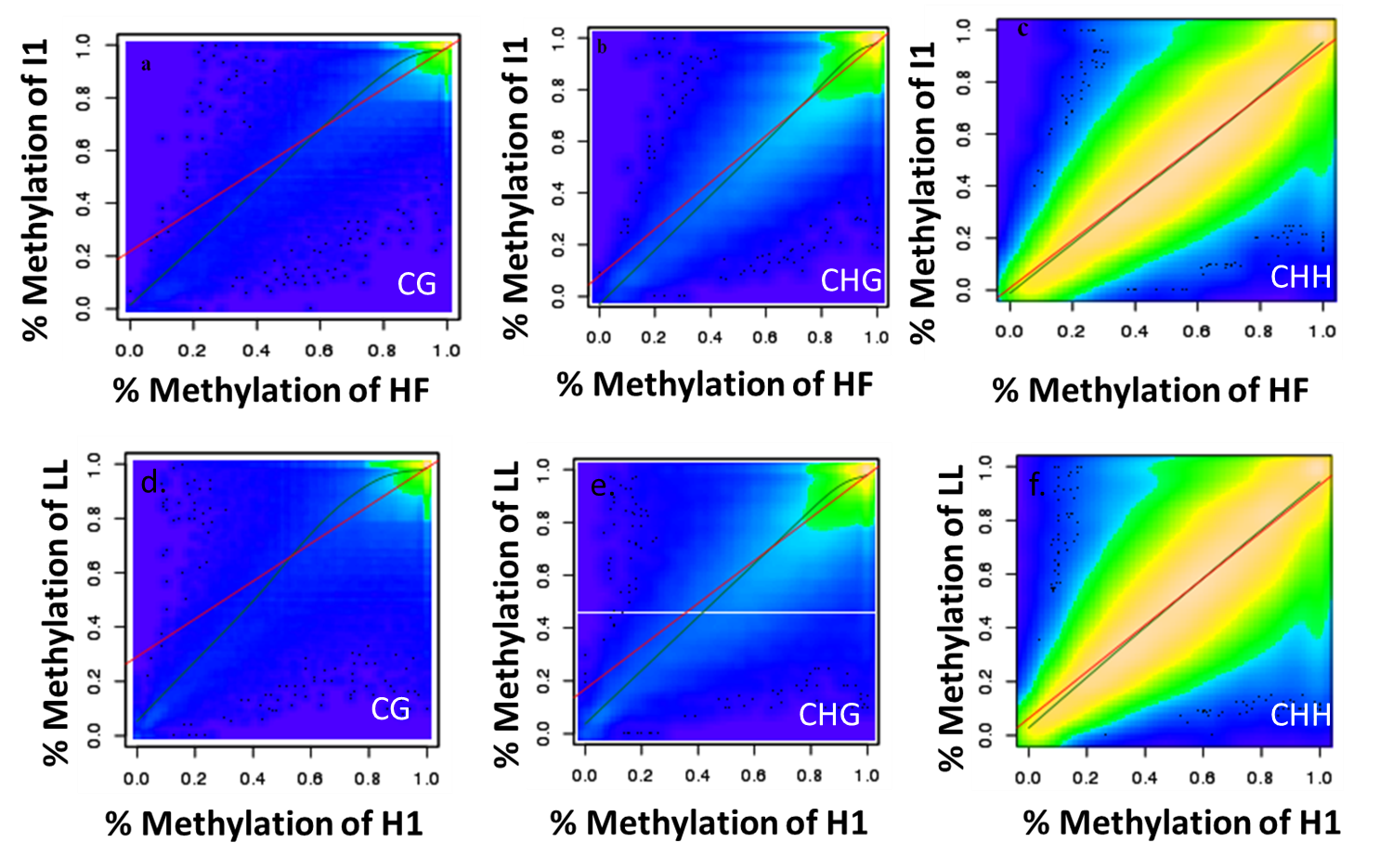

Supplement: Supplementary file 1 [file biology-11-00954-s001.zip › FigureS1.tif]

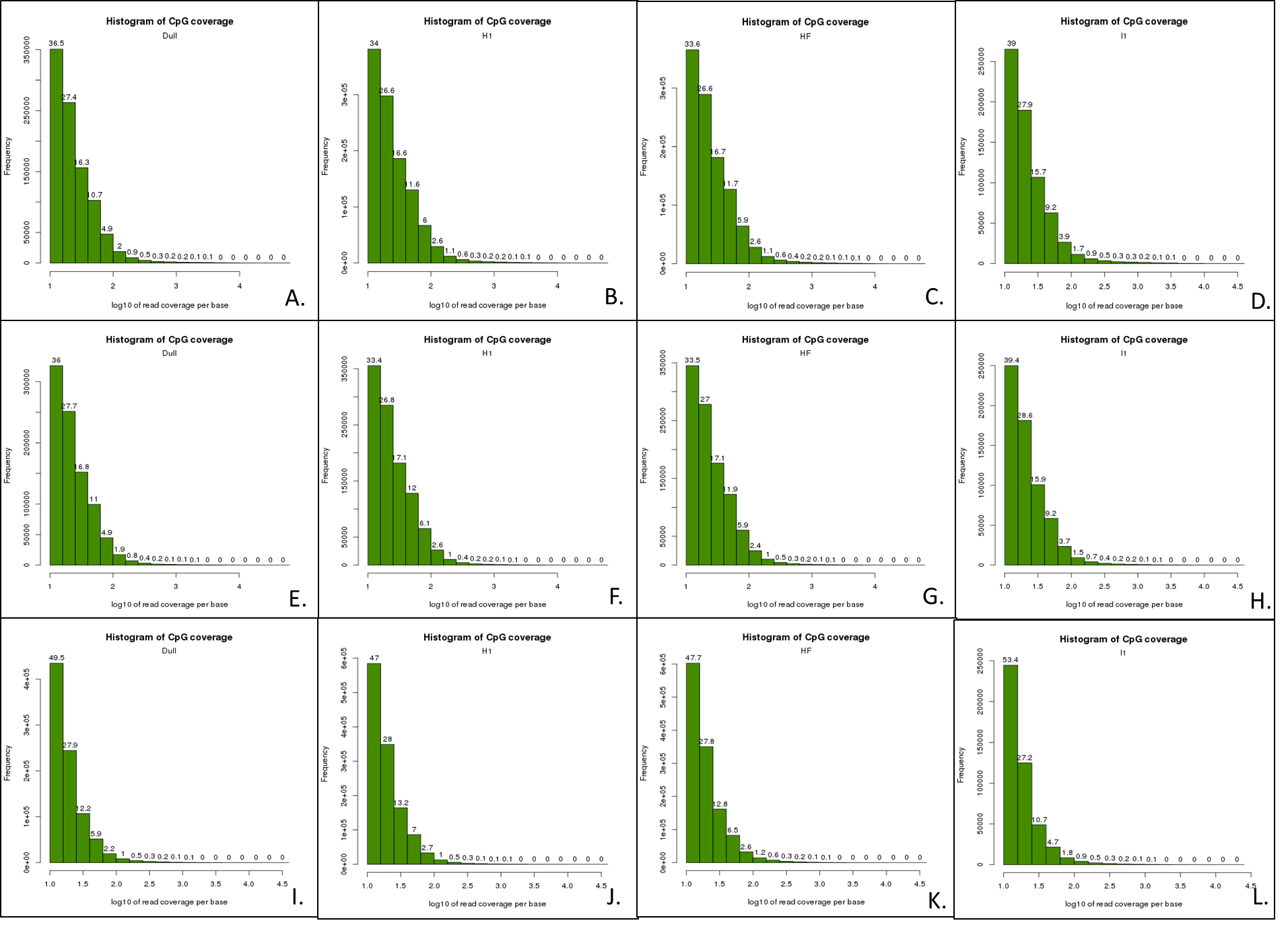

Supplement: Supplementary file 1 [file biology-11-00954-s001.zip › FigureS2.tif]

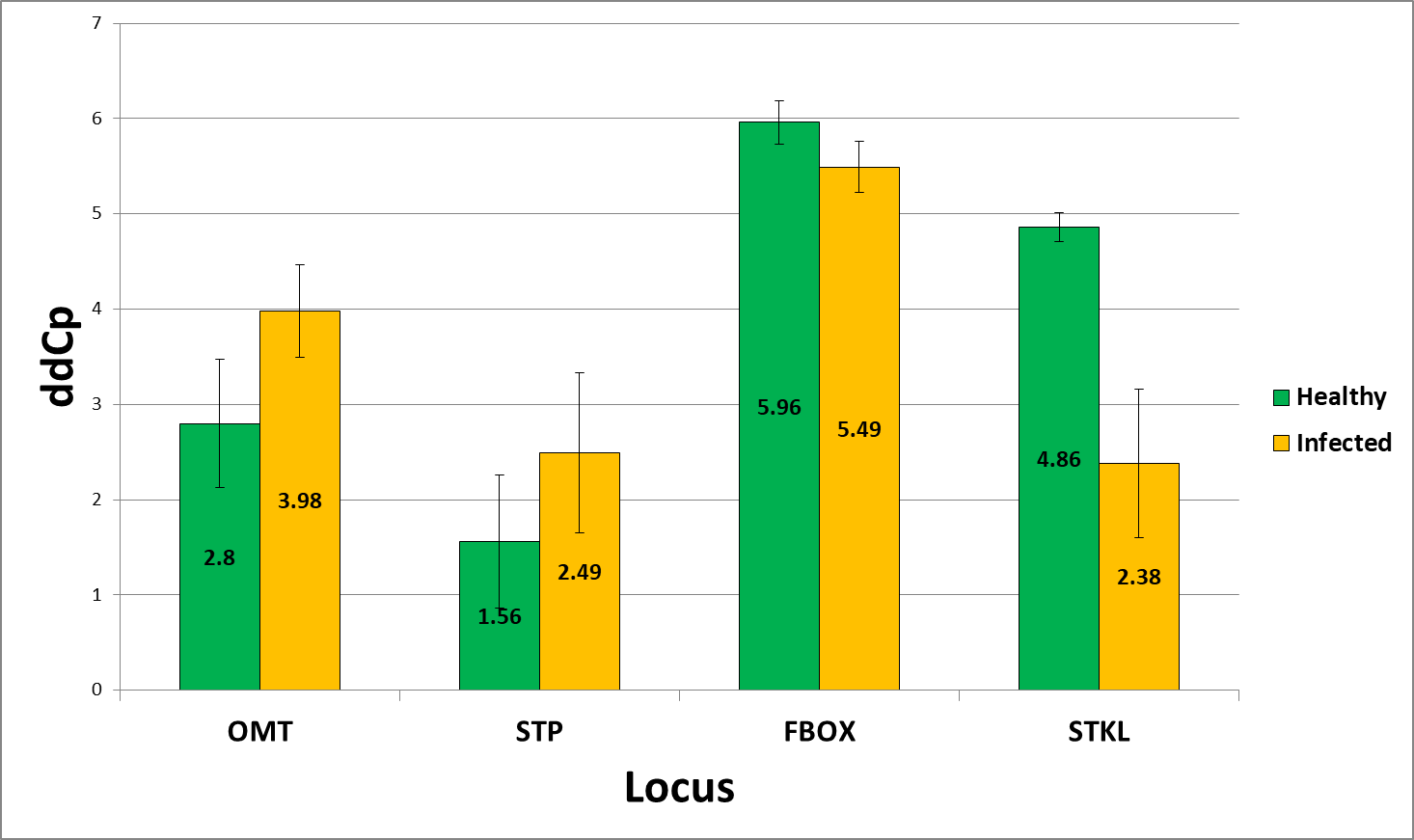

Supplement: Supplementary file 1 [file biology-11-00954-s001.zip › FigureS4.png]

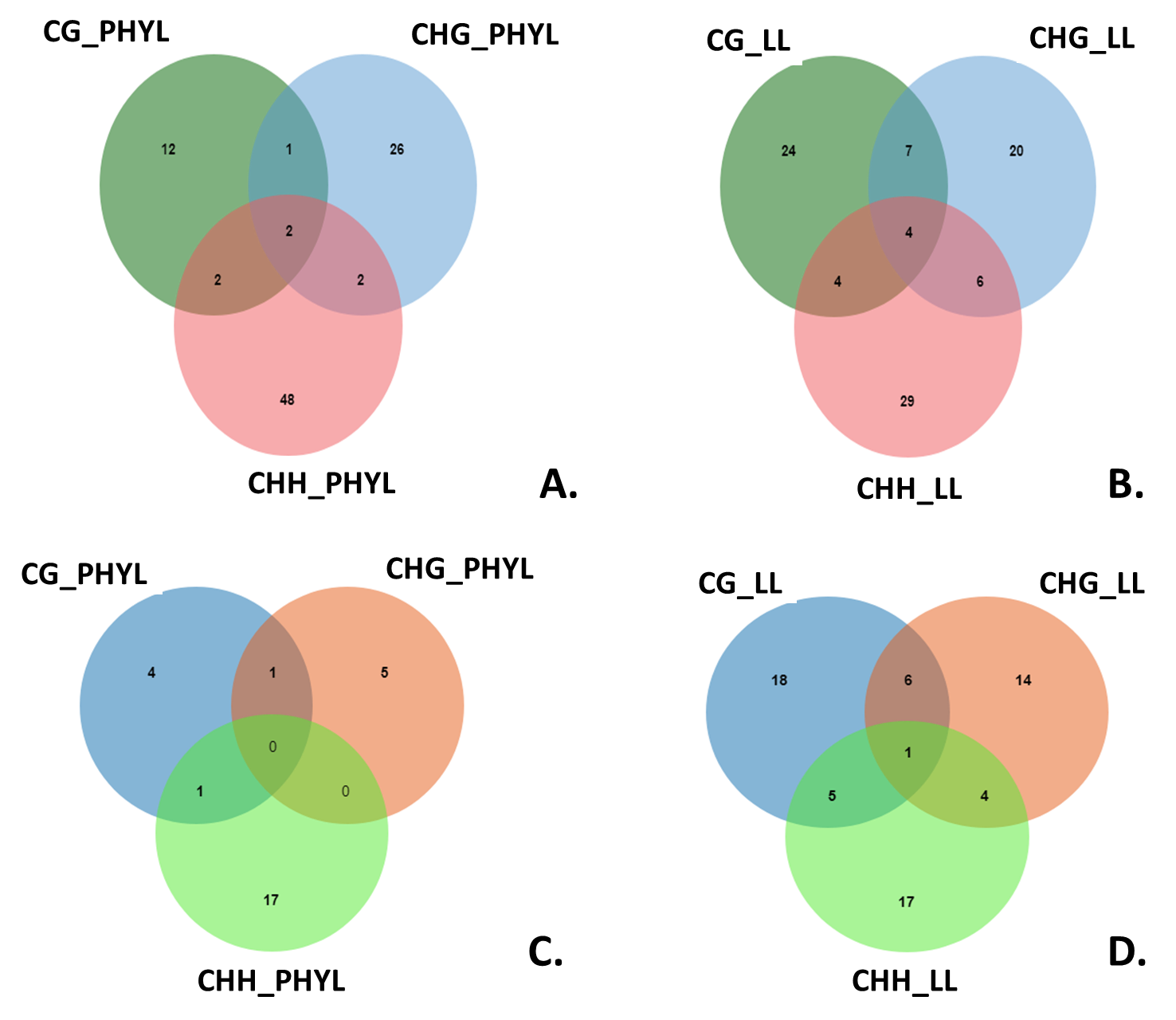

Supplement: Supplementary file 1 [file biology-11-00954-s001.zip › FiguresS3.tif]
